# Metformin Dysregulates the Unfolded Protein Response and the WNT/β-Catenin Pathway in Endometrial Cancer Cells through an AMPK-Independent Mechanism

**DOI:** 10.3390/cells10051067

**Published:** 2021-04-30

**Authors:** Domenico Conza, Paola Mirra, Gaetano Calì, Luigi Insabato, Francesca Fiory, Francesco Beguinot, Luca Ulianich

**Affiliations:** 1 Department of Medical and Translational Sciences & Institute of Endocrinology and Experimental Oncology of CNR, University “Federico II”, 80131 Naples, Italy; domenico.conza@libero.it (D.C.); p.mirra@ieos.cnr.it (P.M.); francesca.fiory@unina.it (F.F.); beguino@unina.it (F.B.); 2 Institute of Endocrinology and Molecular Oncology of CNR, University “Federico II”, 80131 Naples, Italy; g.cali@ieos.cnr.it; 3 Department of Advanced Biomedical Sciences, University “Federico II”, 80131 Naples, Italy; insabato@unina.it

**Keywords:** metformin, endometrial cancer, UPR, AMPK, Wnt/β-catenin

## Abstract

Multiple lines of evidence suggest that metformin, an antidiabetic drug, exerts anti-tumorigenic effects in different types of cancer. Metformin has been reported to affect cancer cells’ metabolism and proliferation mainly through the activation of AMP-activated protein kinase (AMPK). Here, we show that metformin inhibits, indeed, endometrial cancer cells’ growth and induces apoptosis. More importantly, we report that metformin affects two important pro-survival pathways, such as the Unfolded Protein Response (UPR), following endoplasmic reticulum stress, and the WNT/β-catenin pathway. GRP78, a key protein in the pro-survival arm of the UPR, was indeed downregulated, while GADD153/CHOP, a transcription factor that mediates the pro-apoptotic response of the UPR, was upregulated at both the mRNA and protein level. Furthermore, metformin dramatically inhibited β-catenin mRNA and protein expression. This was paralleled by a reduction in β-catenin transcriptional activity, since metformin inhibited the activity of a TCF/LEF-luciferase promoter. Intriguingly, compound C, a well-known inhibitor of AMPK, was unable to prevent all these effects, suggesting that metformin might inhibit endometrial cancer cells’ growth and survival through the modulation of specific branches of the UPR and the inhibition of the Wnt/β-catenin pathway in an AMPK-independent manner. Our findings may provide new insights on the mechanisms of action of metformin and refine the use of this drug in the treatment of endometrial cancer.

## 1. Introduction

Metformin (dimethylbiguanide) is a biguanide drug that is widely used as the first-line pharmacologic treatment of type 2 diabetes. However, data obtained in both cell lines and animal models have shown that metformin, besides its antidiabetic action, exerts also anti-tumorigenic effects [1,2]. Moreover, epidemiological evidence suggests that metformin use lowers cancer risk and reduces the rate of cancer deaths among diabetic patients [3,4,5,6], though lack of effect of metformin in clinical trials has also been reported [7]. Many of both the systemic indirect and direct effects exerted by metformin on cancer cells are thought to be mediated through the activation of the AMP-activated protein kinase (AMPK), a regulator of energy metabolism usually activated in response to cellular stresses that deplete cellular energy levels, increasing the AMP/ATP ratio [8,9,10,11]. AMPK activation, in turn, inhibits the mammalian target of rapamycin (mTOR), thereby reducing both protein synthesis and proliferation of cancer cells. However, the underlying biological mechanisms of metformin antineoplastic activity are still largely unknown and it is becoming increasingly evident that AMPK activation by itself is insufficient to explain the plethora of metformin effects on different types of cancer cells. A number of studies suggests that AMPK activators might exert an anti-cancer effect through the modulation of the unfolded protein response (UPR) in endoplasmic reticulum (ER) stress conditions [12,13,14,15,16,17,18]. The UPR is a survival mechanism of the cell. When cells are exposed to various stressors, the protein folding capacity of the endoplasmic reticulum (ER) is increased, and the overall folding demand of the cell is decreased [19]. If this fails to happen, the ER is “overwhelmed” and apoptotic pathways are activated, leading to cell death [19]. UPR signaling supports malignant transformation and drives tumor survival in the face of increasingly harsh microenvironmental stresses [19]. Consequently, it has been logically hypothesized that targeted UPR inhibitors might also provide an effective anti-cancer approach [20,21,22]. A major UPR effector is the glucose-regulated protein 78 (GRP78), which has important roles in protein folding and assembly, ER Ca^2+^ binding, and controlling the activation of transmembrane ER stress sensors [23]. It has been reported that metformin can downregulate GRP78 expression and trigger the UPR-mediated apoptotic pathway via an AMPK-dependent mechanism in acute lymphoblastic leukemia (ALL) cells [24]. Metformin has also been described to cause transcriptional regulation of UPR in breast cancer cells [25]. Very recently, we have reported that GRP78 overexpression and the UPR activation play important roles in endometrial cancer progression [26,27,28]. However, whether metformin can affect the UPR in endometrial cancer cells and whether this effect might be mediated by AMPK activation is still unknown. In this study we report that metformin is able to affect differentially UPR signaling branches in endometrial cancer cells. The pro-survival UPR protein GRP78 was, indeed, downregulated while the pro-apoptotic axis ATF4/CHOP was upregulated. In addition, we show that metformin is also able to inhibit the Wnt/β-catenin pathway. Wnt/β-catenin signaling has been found to play an essential role in many oncogenic processes in gynecologic malignancies, including tumorigenesis, metastasis, recurrence, and chemotherapy resistance [29]. Interestingly, the effects of metformin on the UPR and the Wnt/β-catenin pathway have recently been described in different cancer cell lines [30]. Thus, metformin, affecting two important pro-survival pathways, might represent an effective drug in the treatment of endometrial cancer, particularly in the presence of high levels of GRP78 and β-catenin expression.

## 2. Materials and Methods

### 2.1. Cell Lines and Reagents

The human endometrial cancer cell lines used were Ishikawa (kindly provided by Prof. Stefania Catalano, University of Calabria, Italy), derived from well-differentiated endometrial adenocarcinoma; HEC1B (kindly provided by Dr. Elvira Crescenzi, IEOS-CNR, Naples, Italy), derived from moderately differentiated endometrial adenocarcinoma; and AN3CA (kindly provided by Dr. Stefania Petrosino, IBC-CNR, Pozzuoli, Italy) derived from undifferentiated endometrial adenocarcinoma. Ishikawa and HEC1B cells were grown in DMEM, AN3CA cells were grown in MEM, and all were supplemented with 10% fetal bovine serum and 300 mM L-glutamine, in a humidified atmosphere with 5% CO_2_. DMEM, MEM, L-glutamine, and FBS were from Lonza (Verviers, Belgium). Metformin (1,1-dimethylbiguanide hydrochloride) was from Sigma-Aldrich (St. Louis, MO, USA) and was dissolved in sterile water and used at the indicated concentration for each specific study. MTT (3-(4,5-dimethylthiazol-2-yl)-2,5-diphenyltetrazolium bromide) dye was purchased from Sigma-Aldrich (St. Louis, MO, USA). The anti-GRP78 antibody has been previously described [26]. Anti-β-actin, anti-PARP, anti-p-Akt, anti-ATF6α, anti-GADD153/CHOP, anti-14.3.3, anti-vinculin, anti lamin A/C, anti-GAPDH, and anti p-GSK3-β antibodies were from Santa Cruz Biotechnologies (Dallas, TX, USA), anti-ATF4 and anti-p-AKT were from Cell Signaling (Danvers, MA, USA), anti p-eIF2α and anti p-AMPK were from Abnova (Taipei, Taiwan), and anti-β-catenin antibodies were from BD Biosciences (San Jose, CA, USA). The enhanced chemiluminescence Western blotting detection reagents were purchased from Pierce Biotechnology (Rockford, IL, USA). Compound C was from Merck Millipore (Darmstadt, Germany). Trizol was from Thermo Fisher Scientific (Waltham, MA, USA).

### 2.2. Cell Proliferation and Colony Formation Assay

In vitro proliferation was assessed with tetrazolium salt 3-(4,5-dimethylthiazol-2-yl)-2,5-diphenyltetrazolium bromide (MTT). Briefly, 5000 cells were plated per well onto 96-well microtiter plates in medium with 10% FBS. After 16 hours metformin was added to the medium for 48 h. Assays were done by incubating each plate with 20 μL of MTT substrate for 2 h followed by removal of medium and addition of 200 μL of dimethylsulfoxide. Plates were read at a wavelength of 570 nm. To determine the long-term inhibitory effects of metformin on cell proliferation, a colony formation assay was conducted. Cells were seeded onto six-well plates at a density of 1000 cells per well. After cell attachment, 5 mM metformin was added or not to the wells for 4 h. The cells were then cultured with fresh medium. After 2 weeks, the resultant colonies were fixed with 4% paraformaldehyde and stained with hematoxylin. The colonies were then photographed and counted under a microscope.

### 2.3. Western Blot

Western blots were carried out as previously reported [26]. Briefly, cells were washed with ice-cold phosphate-buffered saline (PBS) and harvested in Laemmli buffer (with β-mercaptoethanol) containing a mixture of phosphatase inhibitors (0.5 mM sodium vanadate, 2 mM sodium pyrophosphate, 5 mM β-glycerolphosphate, and 50 mM sodium fluoride) to prevent post-lysis dephosphorylation. After evaluation of protein content, 30 μg of cell extract was analyzed by SDS-PAGE and electrotransferred to polyvinylidene difluoride. Blocking was for 15 h at 4 °C with Tris-buffered saline-Tween 20 (TBST) buffer (10 mM Tris (H 8.0), 150 mM NaCl, 0.1% Tween 20) containing 5% bovine serum albumin, followed by incubation in TBST buffer for 2 h at room temperature with a 1:2000 dilution of anti-GRP78, anti-14.3.3, and anti-β-actin, 1:1000 anti-lamin A/C, 1:2000 anti-vinculin, 1:2000 anti-GAPDH, 1:500 anti-p-eIF2α, 1:1000 anti-PARP, anti-ATF4, anti-ATF6, anti-CHOP, anti-p-AKT, anti-p-AMPK, anti-p-S6-kinase, and anti-β-catenin. After being washed with TBST, the blot was incubated for 1 h at room temperature with anti-rabbit horseradish peroxidase-conjugated antibodies diluted 1:3000 in TBST. Band detection was by enhanced chemiluminescence. Densitometric analysis was performed on a Macintosh computer using the public domain NIH Image J program (developed at the U.S. National Institutes of Health; http://rsb.info.nih.gov/nih-image/ accessed on 29 April 2021).

### 2.4. RNA Isolation and Real-Time Reverse Transcription-PCR

Total RNA was extracted with the TRIzol reagent, according to the manufacturer’s protocol. Reverse transcription of 1 μg of total RNA was performed using SuperScript III, following the manufacturer’s instructions. Quantitative real-time RT-PCR analysis was performed as previously described [26]. Briefly, reactions were performed in triplicate by using iQ SYBR Green Supermix on iCycler real-time detection system (Biorad, Hercules, CA, USA). Relative quantification of gene expression was calculated by the ΔΔCt method. Each Ct value was first normalized to the respective glyceraldehyde-3-phosphate dehydrogenase (GAPDH) Ct value of a sample to account for variability in the concentration of RNA and in the conversion efficiency of the RT reaction. Oligonucleotides used were: 5′-aggcttatcttccttcagtggc-3′ and 5′-cgctctctgctcctcctgttc-3′ for GAPDH; 5′-ttgactccgaccttcaccttcc-3′ and 5′-tttcacagtggccaagagtc-3′ for BIP/GRP78; 5′-agcatgttcctgaggagttgg-3′ and 5′-aggcttatcttccttcagtggc-3′ for ATF6; 5′-gcacctcccagagccctc actctcc-3′ and 5′-gtctactccaagccttccccctgcg-3 for CHOP/GADD153; 5’-tcaaacctcatgggt tctcc-3’ and 5’-gtgtcatccaacgtggtcag-3’ for ATF4; and 5’- gctttcagttgagctgacca-3’ and 5’-caagtccaagatcagcagtctc-3’ for β-catenin.

### 2.5. Wound Healing Assay

Cells (1 × 10^6^ per well) were seeded in six-well plates and allowed to adhere for 24 h. Confluent monolayer cells were scratched by a 200 μL pipette tip and then washed three times with 1× PBS to clear cell debris and suspension cells. Fresh medium containing or not the different stimuli was added, and the cells were allowed to close the wound for 48 h. Photographs were taken at 0 and 48 h at the same position of the wound.

### 2.6. Luciferase Assay

For transient transfection analysis, cells were plated in 6-well plates to approximately 70% confluence 24 h before transfection. Cells were transfected with 1.0 μg of the reporter vector BAT-LUX TCF/LEF (kindly provided by Prof. G. Viola, University of Padova, Italy) and 50 ng of pRL-TK vector (Promega, Madison, WI, USA) with Lipofectamine 3000 (Invitrogen, Carlsband, CA, USA) according to manufacturer instructions. BAT-LUX drives the expression of a firefly luciferase gene under the control of seven TCF/LEF binding sites in the backbone of pGL3. After 24 h, transfection medium was replaced with fresh medium and cells were treated or not for 24 h with 5 mM metformin. Finally, firefly and renilla activities were determined in cell lysates using the Dual-Luciferase Reporter Assay System (Promega, Madison, WI, USA) and a luminometer (Orion I, Berthold Detection Systems, Baden Württemberg, Germany) according to the manufacturer’s instructions. Results were expressed as the ratio of firefly to renilla activity.

### 2.7. Immunofluorescence

1.5 × 10^5^ cells were plated on 12 mm diameter glass coverslips. Forty-eight hours later, cells were vehicle-treated or treated with 5 mM metformin for 24 h. Immunofluorescence was performed as previously reported [31]. Briefly, cells were fixed in 4% paraformaldehyde in PBS for 20 min, washed twice in 50 mM NH4Cl in PBS, and permeabilized for 5 min in 0.1% Triton X-100 in PBS. Nuclei were stained with HOECHST 33258. Immunofluorescence analysis was performed on an inverted, motorized microscope (Axio Observer Z.1) equipped with a 63X/1.4 Plan-Apochromat objective (Carl Zeiss, Gottingen, Germany). The attached laser-scanning unit (LSM 700 4X pigtailed laser 405-488-555-639, Carl Zeiss, Gottingen, Germany) enabled confocal imaging. For excitation, 405 and 555 nm lasers were used. Fluorescence emission was revealed by a MBS (Main Dichroic Beam Splitter) and a VSD (Variable Secondary Dichroic Beam Splitter). Double staining fluorescence images were acquired separately using ZEN 2012 software in the blue (Hoechst 33258), and red (Alexa Fluor 594) channels at a resolution of 1024 × 1024 pixels, with the confocal pinhole set to one Airy unit, and then saved in TIFF format.

### 2.8. Cell Fractionation

Cells were lysed in hypotonic buffer (20 mM Tris–HCl pH 7.4, 10 mM NaCl, 3 mM MgCl2 protease, and phosphatase inhibitor mixture solution) for 15 min at 4 °C. After centrifugation at 850× *g* for 10 min, supernatants were collected to obtain the cytoplasmic proteins. The nuclear pellets were resuspended in extraction buffer (100 mM Tris Ph 7.4, 100 mM NaCl, 1 mM EDTA, 1 mM EGTA, 0.1% SDS, 1% Triton X100, 0.5% Deoxycholate, protease, and phosphatase inhibitor mixture solution) for 30 min at 4 °C with vortexing. After centrifugation at 10,000× *g* for 30 min the supernatants were collected.

### 2.9. Statistical Analysis

Each experiment was performed at least three times, data are shown as the mean ± SD where applicable, and differences were evaluated using one-way ANOVA for 3-group comparisons and *t* tests for 2-group comparisons. All statistical analyses were performed using SPSS 13.0 software package. The probability of *p* < 0.05 was considered to be statistically significant.

## 3. Results

### 3.1. Metformin Affects Cell Growth and Viability of Endometrial Cancer Cells

**Figure 1:**
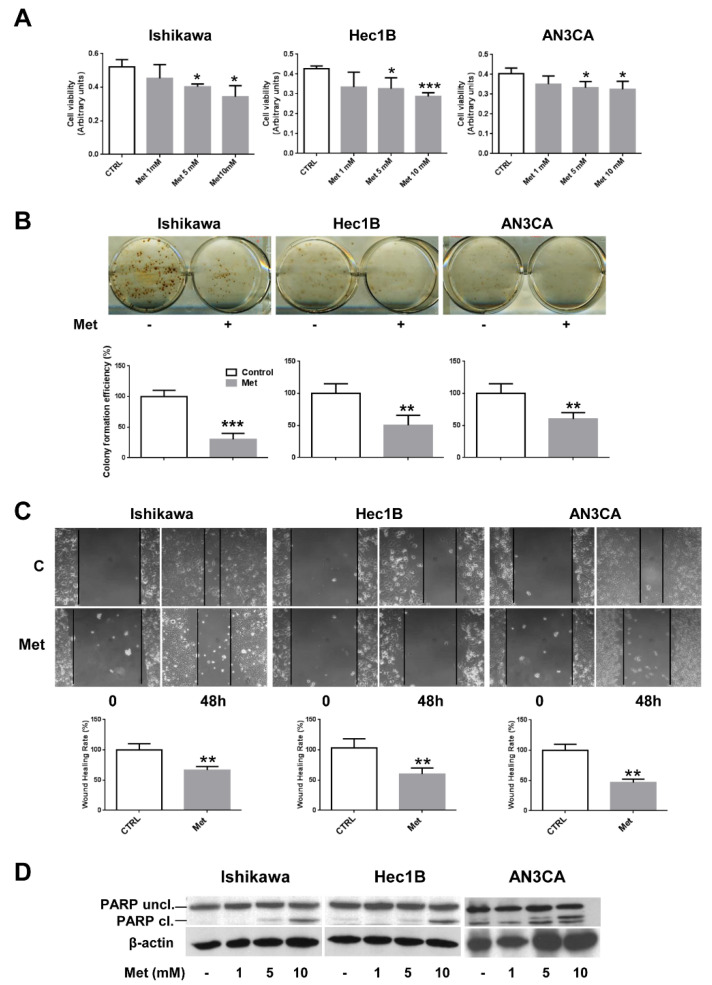
Metformin inhibits cell growth and viability in endometrial cancer cells. (**A**) Ishikawa, HEC1B, or AN3CA cells were seeded at a density of 5 × 10^3^ cells in a 96-well plate. After 16 h, cells were treated or not with 1, 5, or 10 mM metformin. Cell viability was measured after 48 h using the MTT assay. Values represent the mean absorbance at 570 nm ± SD of triplicates of three independent experiments. * indicates a *p*-value < 0.05; *** indicates a *p*-value < 0.001. (**B**) Ishikawa, HEC1B, or AN3CA cells were seeded onto six-well plates at a density of 1 × 10^3^ cells per well. After cell attachment, 5 mM metformin was added or not to the wells for 4 h. The cells were then cultured with fresh medium. After 2 weeks, the resultant colonies were fixed with 4% paraformaldehyde and stained with hematoxylin. The colonies were then photographed, counted under a microscope, and colony efficiency formation was calculated. ** indicates a *p*-value < 0.01; *** indicates a *p*-value < 0.001. (**C**) Ishikawa, HEC1B, or AN3CA cells (1 × 10^6^ per well) were seeded in six-well plates and allowed to form a cell monolayer for 24 h. Cell layers were wounded with a micropipette tip and then incubated in fresh culture medium containing or not 5 mM metformin for 48 h. Cell migration toward the wounded area was observed, photographed, and measured. Experiments were performed three times in triplicate. Graphs show the percentage of wound healing rate. ** indicates a *p*-value < 0.01. (**D**) Ishikawa, HEC1B, or AN3CA cells were treated or not with 1, 5, or 10 mM metformin for 48 h. Total cellular proteins were extracted and Western blot experiments were performed with antibodies against PARP and β-actin.

Since it has been recently reported that metformin is able to inhibit the cell growth and viability of well-differentiated endometrial cancer cells, such as Ishikawa and ECC-1 [32,33,34], we sought to evaluate whether metformin was able to affect the proliferation of endometrial adenocarcinoma cell lines owing different features. To this aim, besides Ishikawa cells, we performed an MTT assay also on HEC1B (a moderately differentiated cell line) and AN3CA cells (a poorly differentiated cell line) treated with different concentrations of metformin. As shown in Figure 1A, metformin inhibited significantly the proliferation of all the cell lines tested in a dose-dependent manner, albeit with different efficacy, when compared to untreated cells. Next, we utilized two-dimensional clonogenic survival assay to detect the cells’ capability for colony formation after metformin (5 mM) treatment. The results show that metformin could significantly inhibit colony formation in all the cell lines tested (Figure 1B). Wound-healing assays were also performed to investigate the potential inhibitory effect of metformin on cell migration of endometrial cancer cell lines. Therefore, Ishikawa, HEC1B, and AN3CA cells were incubated in serum-free media containing 5 mM metformin for 48 h. As shown in Figure 1C, the results of the wound-healing assays suggest that the migration of endometrial cancer cells was inhibited by metformin when compared to untreated cells. Finally, to assess whether metformin could cause apoptosis in endometrial cancer cells, we analyzed PARP cleavage by Western blot experiments. Cleavage of PARP by caspases is considered to be, indeed, a hallmark of apoptosis [35]. As shown in Figure 1D and Supplementary Materials Figure S3, the proteolytic cleavage of PARP (PARP Cl.) was detectable at the concentrations of 5 and 10 mM in all the endometrial cancer cell lines, suggesting that metformin induces apoptosis in endometrial cancer cells.

### 3.2. CC Does Not Prevent Metformin Effects on Endometrial Cancer Cells

**Figure 2:**
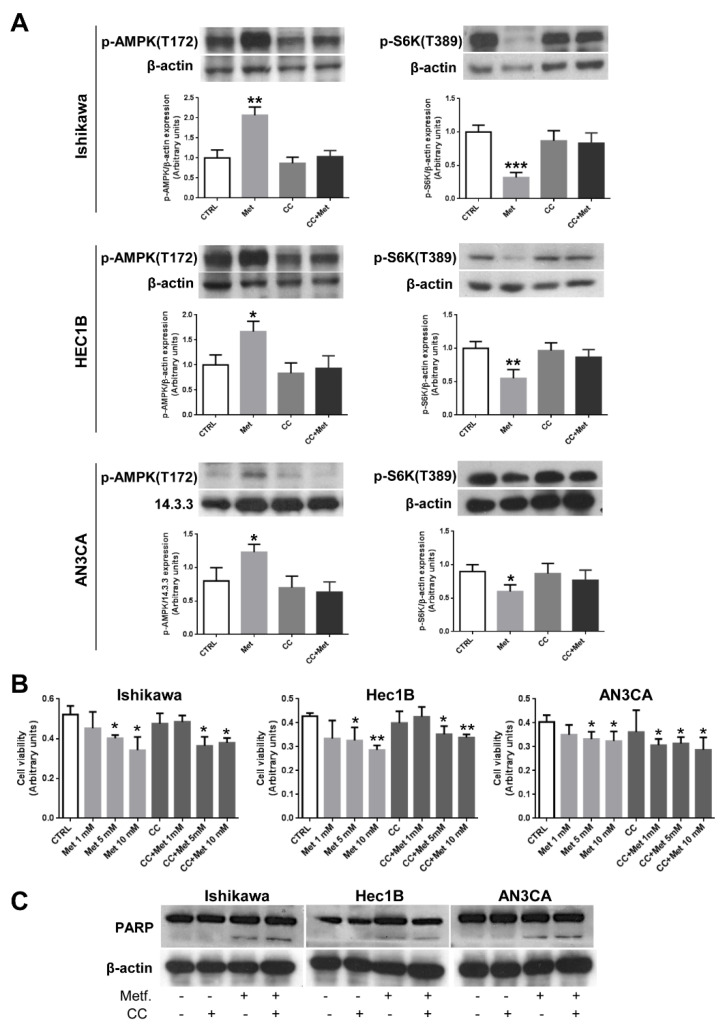
Inhibition of AMPK by CC does not alter metformin effects on endometrial cancer cells. (**A**) Ishikawa, HEC1B, or AN3CA cells were treated or not for 24 h with 5 mM metformin or 10 µM CC or pretreated for 1 h with 10 µM CC followed by treatment with 5 mM metformin. Total cellular proteins were extracted and Western blot experiments were performed, as described in the Section 2.3, using antibodies against p-AMPK (left panels) or p-S6 kinase (right panels). Data represent the mean ± SD of three independent experiments. * *p* < 0.1; ** *p* < 0.05; *** *p* < 0.01. (**B**) Ishikawa, HEC1B, or AN3CA cells were seeded at a density of 5 × 10^3^ cells in a 96-well plate. After 16 h, cells were treated or not with increasing concentration of metformin in the presence or absence of 1 h pretreatment with 10 µM CC. Cell viability was measured after 48 h using the MTT assay. Values represent the mean absorbance at 570 nm ±SD of triplicates of three independent experiments. * indicates a *p*-value < 0.05; ** indicates a *p*-value < 0.01. (**C**) Ishikawa, HEC1B, or AN3CA cells were treated or not for 48 h with 5 mM metformin or 10 µM CC or pretreated for 1 h with 10 µM CC followed by treatment with 5 mM metformin. Total cellular proteins were extracted and Western blot experiments were performed, as described in the Section 2.3, with antibodies against PARP or β-actin (loading control).

Metformin has already been described to activate AMPK and decrease mTOR signaling in well-differentiated endometrial cancer cells [33,36]. Since we observed that metformin affected endometrial cancer cells’ growth and viability regardless of their degree of differentiation, we evaluated whether metformin was also able to activate AMPK at a similar extent. As shown in Figure 2A (left panels), AMPK phosphorylation increased significantly, following metformin treatment, in all cell lines. As expected, pretreatment of cells with the well-known AMPK inhibitor CC prevented almost completely AMPK activation (Figure 2A, left panels). Accordingly, with the activation of the AMPK signaling cascade, we observed the inhibition of mTOR signaling measured by phosphorylated S6 protein kinase (Figure 2A, right panels). Again, CC was able to prevent S6 kinase dephosphorylation (Figure 2A, right panels) and, thus, mTOR inhibition, in all endometrial cancer cell lines. Therefore, metformin activates AMPK and CC is able to prevent almost completely AMPK activation in endometrial cancer cells. Since it has been described that the anti-cancer effects exerted by metformin are mainly mediated by AMPK activation [8,9,10,11], we performed MTT assays on Ishikawa, HEC1B, and AN3CA cells treated with metformin in the presence of CC. However, as shown in Figure 2B, CC was not able to prevent significantly the inhibition of proliferation caused by metformin treatment. Furthermore, CC was ineffective also in preventing PARP cleavage induced by metformin treatment (Figure 2C), suggesting the existence of other mechanism/s, besides AMPK activation, playing a role in the anti-cancer effect of metformin on endometrial cancer cells.

### 3.3. Metformin Modulates the Expression of UPR Key Players in Endometrial Cancer Cells

**Figure 3:**
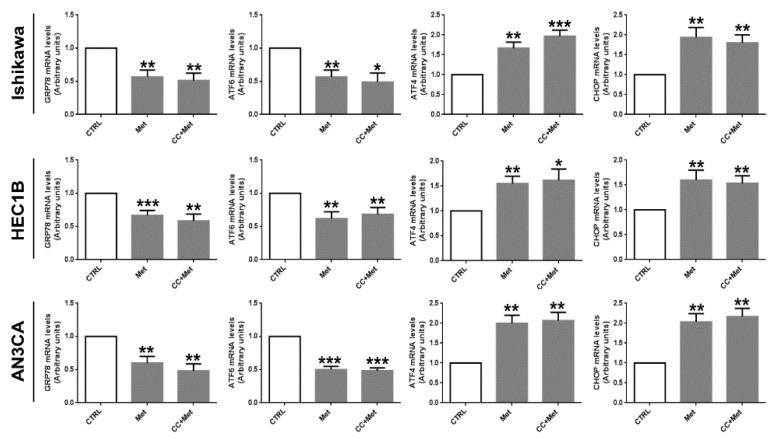
Metformin modulates the mRNA expression of UPR genes in an AMPK-independent manner in endometrial cancer cells. Ishikawa, HEC1B, or AN3CA cells were treated or not for 24 h with 5 mM metformin in the presence or absence of 1 h pretreatment with 10 µM CC. Total RNA was extracted and real-time RT-PCR experiments were performed using oligonucleotides specific for GRP78, ATF6, ATF4, CHOP, and GAPDH as described in the Section 2.4. Values shown represent the mean (± s.d.) of triplicate samples of three independent experiments. * *p* < 0.1; ** *p* < 0.05; *** *p* < 0.01.

Since AMPK activators have been recently reported to exert anti-cancer effects through ER stress induction [12,24,37], suggesting an association between AMPK activation and ER stress, we sought to investigate, first of all, whether metformin might affect the UPR in endometrial cancer cells. To this purpose we performed real-time RT-PCR experiments. As shown in Figure 3, metformin was able to modify the mRNA expression of key genes involved in the UPR. In particular, GRP78, whose expression is generally upregulated following ER stress and that is associated with pro-survival responses [28], was significantly downregulated, while mRNA expression of GADD153/CHOP, a transcription factor that mediates the UPR-dependent apoptotic response, was markedly upregulated. Interestingly, pretreatment of cells with CC did not prevent metformin effect on mRNA expression of UPR genes (Figure 3). 

**Figure 4:**
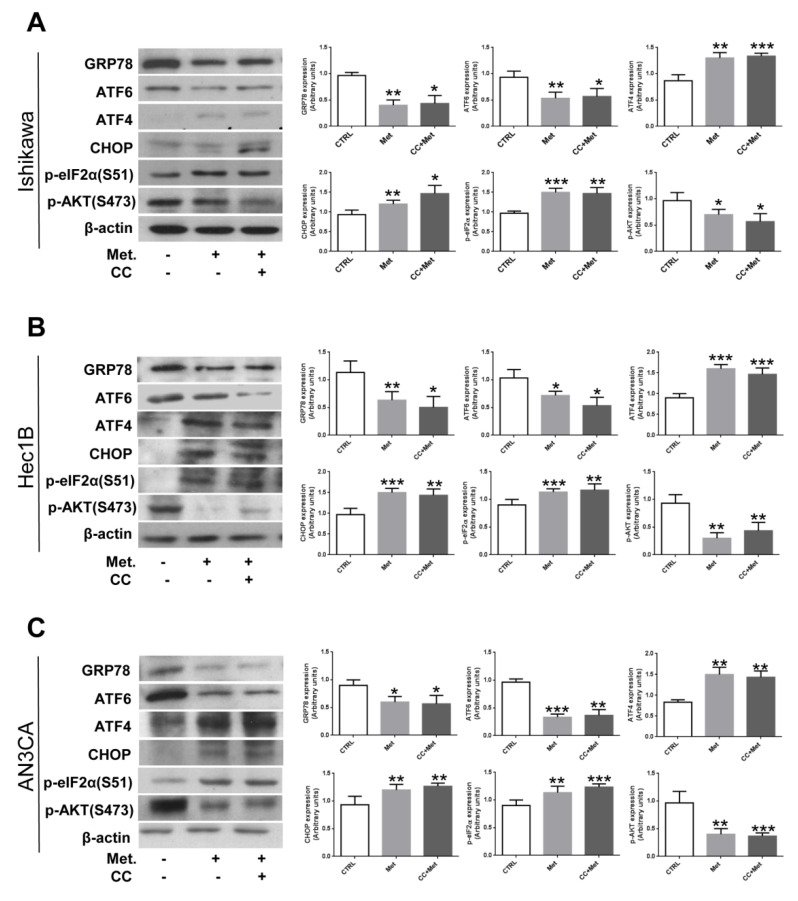
Metformin modulates the expression/phosphorylation of UPR proteins and AKT in an AMPK-independent manner in endometrial cancer cells. Ishikawa (**A**), HEC1B (**B**), or AN3CA (**C**) cells were treated or not for 24 h with 5 mM metformin in the presence or absence of 1 h pretreatment with 10 µM CC. Total cellular proteins were extracted and Western blot experiments were performed with antibodies against GRP78, ATF6, ATF4, p-eIF2α, *p*-AKT, or β-actin (loading control), as described in the Section 2.3. Values shown represent the mean (± s.d.) of three independent experiments. * *p* < 0.1; ** *p* < 0.05; *** *p* < 0.01.

Next, we analyzed metformin’s effect on UPR protein expression or phosphorylation. As shown in Figure 4 and Supplementary Materials Figure S1, metformin treatment affected GRP78, CHOP, ATF6, and ATF4 protein expression levels similarly to mRNA expression. In addition, we show that the phosphorylation of the translation initiation factor eIF2α, a direct substrate of PERK, was significantly increased, confirming the activation of the PERK/ATF4/CHOP axis. In addition, since it has been recently reported that inhibiting GRP78 expression by the use of specific siRNAs might affect AKT activity and sensitize endometrial cancer cells to apoptosis [37], we evaluated AKT phosphorylation upon metformin treatment. As shown in Figure 4 and Supplementary Materials Figure S1, AKT phosphorylation was significantly reduced in endometrial cancer cell lines following metformin treatment. Again, pretreatment of cells with CC did not modify significantly metformin’s effect on the analyzed proteins’ expression or phosphorylation. These data suggest that metformin is able to affect differentially UPR signaling branches in endometrial cancer cells, and that this effect appears to be independent from AMPK activation.

### 3.4. Metformin Inhibits β-Catenin Expression and Activity in Endometrial Cancer Cells

**Figure 5:**
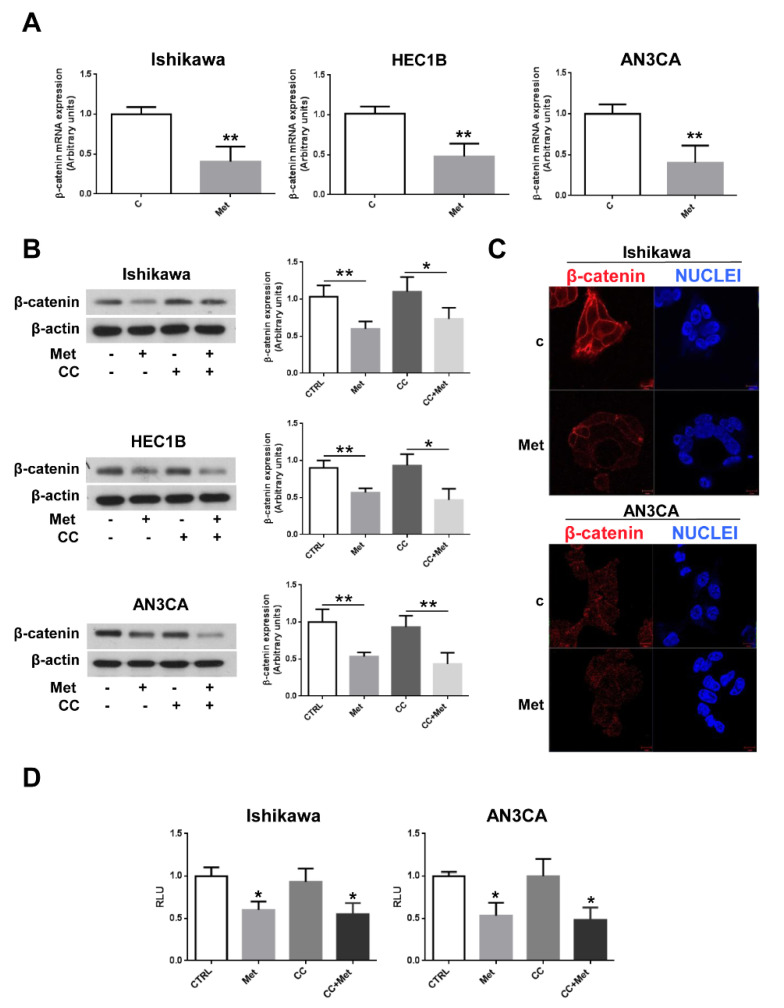
Metformin inhibits β-catenin expression independently from AMPK activation in endometrial cancer cells. (**A**) Ishikawa, HEC1B, or AN3CA cells were treated or not for 24 h with 5 mM metformin. Total RNA was extracted and real-time RT-PCR experiments were performed using oligonucleotides specific to β-catenin and GAPDH as described in the Section 2.4. Values shown represent the mean (± s.d.) of triplicate samples of three independent experiments. ** *p* < 0.05. (**B**) Ishikawa, HEC1B, or AN3CA cells were treated or not for 24 h with 5 mM metformin in the presence or absence of 1 h pretreatment with 10 µM CC. Total cellular proteins were extracted and Western blot experiments were performed with antibodies against β-catenin or β-actin (loading control), as described in the Section 2.3. Values shown represent the mean (± s.d.) of three independent experiments. * *p* < 0.1; ** *p* < 0.05. (**C**) Ishikawa and AN3CA cells were grown on glass coverslips for 48 h, then were treated or not for 24 h with 5 mM metformin. Cells were fixed in 4% paraformaldehyde in PBS for 20 min, washed twice in 50 mm NH4Cl in PBS, and permeabilized for 5 min in 0.1% Triton X-100 in PBS. Cells were double-stained with anti-β-catenin antibodies and HOECHST 33258 (Nuclei). Bars, 10 μm. (**D**) Ishikawa and AN3CA cells were plated in six-well plates to approximately 80% confluence 24 h before transfection. Cells were then transfected with 1.0 μg of the reporter vector BAT-LUX TCF/LEF and 50 ng of pRL-TK vector with Lipofectamine 3000. After 24 h, transfection medium was replaced with fresh medium and cells were treated or not for 24 h with 5 mM metformin, 10 µM CC, or pretreated for 1 h with 10 µM CC followed by treatment with 5 mM metformin. Firefly and renilla activities were determined in cell lysates using the Dual-Luciferase Reporter Assay System and a luminometer. Results were expressed as the ratio of firefly to renilla activity. Values shown represent the mean (± s.d.) of triplicate samples of three independent experiments. * *p* < 0.1.

The most pronounced metformin anti-cancer effects have been reported in colorectal adenocarcinoma where Wnt/β-catenin signaling is frequently aberrantly activated [37,38]. Thus, since Wnt/β-catenin signaling has been found to play an essential role also in gynecologic malignancies [29], we sought to verify whether metformin, beside its capability to modulate UPR, could also inhibit the Wnt/β-catenin signaling in endometrial cancer cells. As shown by real-time RT-PCR experiments, β-catenin mRNA expression was significantly downregulated in Ishikawa, HEC1B, and AN3CA cells (Figure 5A). Next, we analyzed β-catenin protein expression upon metformin treatment. As shown in Figure 5B and in Supplementary Materials Figure S2, metformin caused a dramatic inhibition of β-catenin protein expression levels. Intriguingly, the inhibition of AMPK activation by CC did not affect metformin’s ability to inhibit β-catenin protein expression (Figure 5B), suggesting that, at variance with colon cancer cells [39], metformin’s effect on the Wnt/β-catenin pathway, analogously to what was observed for UPR, was AMPK-independent. The dramatic effect of metformin on β-catenin expression was well documented also by immunofluorescence experiments (Figure 5C). To assess whether metformin, beside β-catenin expression, could affect the Wnt/β-catenin signaling cascade, we performed luciferase assays on Ishikawa and AN3CA cells transfected with the reporter vector BAT-LUX that drives the expression of the firefly luciferase gene under the control of the TCF/LEF promoter, known to be regulated by β-catenin. As shown in Figure 5D, metformin treatment inhibited significantly TCF/LEF transcriptional activity and, thus, Wnt/β-catenin signaling in endometrial cancer cells.

### 3.5. Metformin Inhibits GSK3β Phosphorylation in Endometrial Cancer Cells

**Figure 6:**
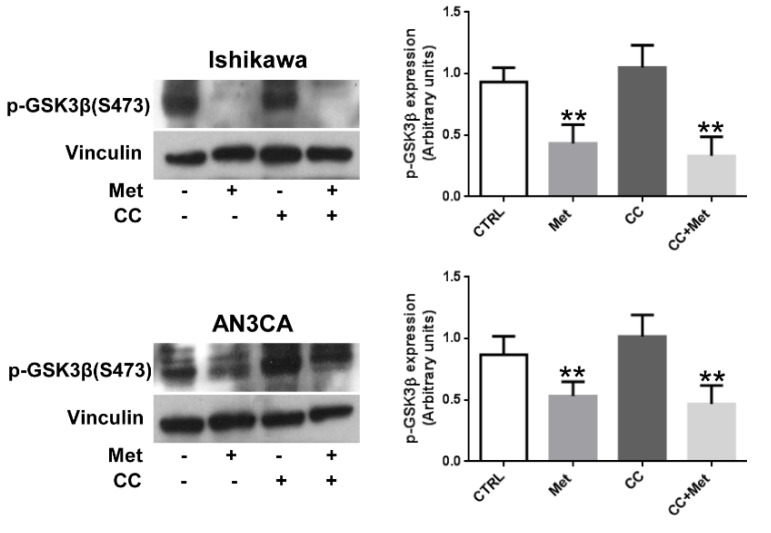
Metformin inhibits GSK3β phosphorylation in endometrial cancer cells. Ishikawa or AN3CA cells were treated or not for 24 h with 5 mM metformin or 10 µM CC or pretreated for 1 h with 10 µM CC followed by treatment with 5 mM metformin. Total cellular proteins were extracted and Western blot experiments were performed with antibodies against p-GSK3β (Ser9) or vinculin (loading control), as described in the Section 2.3. Values shown represent the mean (± s.d.) of three independent experiments. ** *p* < 0.05.

Finally, since β-catenin is well known to be mainly regulated at a posttranslational level by GSK-3β phosphorylation, we sought to assess whether metformin, beside its capability to inhibit β-catenin expression, might also affect β-catenin protein stability. As shown in Figure 6, metformin treatment was able to reduce significantly GSK3β (Ser9) phosphorylation, suggesting that metformin can affect both β-catenin expression and stability in endometrial cancer cells. Interestingly, CC, as observed for AKT, was ineffective in preventing GSK3β phosphorylation.

## 4. Discussion

**Figure 7:**
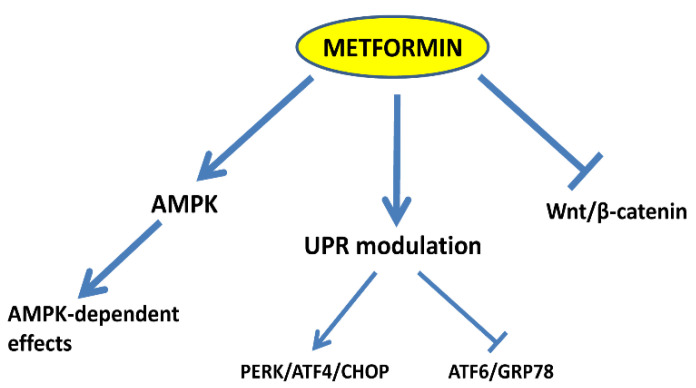
Scheme summarizing the observed mechanisms affected by metformin in endometrial cancer cells. Besides AMPK activation, metformin modulates the UPR by activating the PERK/ATF4/CHOP axis and inhibiting the ATF6/GRP78 axis. Furthermore, metformin inhibits the Wnt/β-catenin signaling pathway by reducing β-catenin expression.

A number of molecular mechanisms have been proposed to explain the anti-cancer activity of metformin [33,40,41,42,43,44,45,46]. Among these, the mechanism that has been more extensively investigated is certainly that linked to metformin’s ability to inhibit oxidative phosphorylation (OXPHOS) at the mitochondrial level, ultimately leading to an increase in the AMP/ATP ratio and to AMPK activation [8,9,10,11,47]. However, many aspects of metformin antineoplastic activity are still largely unknown and it is becoming increasingly evident that AMPK activation by itself is insufficient to explain the plethora of metformin effects on different types of cancer cells. Here, we report for the first time that metformin, beside AMPK, is able to affect two molecular pathways that play important roles in endometrial cancer, such as the UPR and the Wnt/β-catenin pathway. In particular, we show that metformin reduces the growth and induces apoptosis of endometrial cancer cells (Figure 1). These effects were associated, as expected, with the activation of AMPK and with the reduction in S6 protein kinase phosphorylation (Figure 2A) and, thus, with the inhibition of mTOR activity. However, CC, which was effective in preventing the phosphorylation of AMPK by metformin (Figure 2A), was ineffective in significantly modifying metformin effects on both endometrial cancer cells’ proliferation and viability (Figure 2B,C). Intriguingly, we found that metformin modulates the expression of key players of the UPR at both the mRNA and protein level (Figure 3 and Figure 4, respectively). In particular, GRP78 and ATF6 were downregulated while ATF4 and CHOP expression and p-eIF2α phosphorylation were upregulated. This is of relevance, since the ATF6/GRP78 axis usually promotes cell survival while the ATF4/CHOP axis is mainly involved in pro-apoptotic responses [19]. We have recently shown that GRP78 is overexpressed in endometrial cancer tissues and that this protein plays an important role in the growth and invasiveness of endometrial cancer cells [26,27,28]. Accordingly, attenuating GRP78 expression in these cells by the use of specific shRNAs, we observed a reduction in both proliferation and invasion capability [26,27]. Thus, metformin might contribute to shift the balance between cell survival and apoptosis by modulating differentially the UPR signaling branches. This appears not to be mediated by AMPK in endometrial cancer cells, since preventing its activation by CC did not modify significantly metformin’s action on UPR gene expression (Figure 3 and Figure 4 and Supplementary Materials Figure S1). The effects of metformin on the UPR have been described by several studies. Generally, metformin has been reported to inhibit the UPR [12,13,14,15,16,17,24,25,37,45]. However, this action does not necessarily appear to be mediated by AMPK activation, probably depending on the cellular context. A very recent study shows, indeed, that inhibition of AMPK by CC does not alter the effect of metformin on the expression levels of ER stress markers (ATF4, CHOP, FKBP11, and GRP94) in pancreatic β-cells exposed to palmitate [46]. On the contrary, metformin can downregulate GRP78 expression and trigger the UPR-mediated apoptotic pathway via an AMPK-dependent mechanism in acute lymphoblastic leukemia (ALL) cells [24]. Again, metformin sensitizes the anti-cancer effect of dasatinib in head and neck squamous cell carcinoma cells through AMPK-dependent ER stress [14]. Besides UPR modulation, we observed also that metformin reduced significantly Akt activity (Figure 4 and Supplementary Materials Figure S1), probably enhancing the pro-apoptotic effects of ATF4/CHOP branch activation. Accordingly, reduced Akt activity and sensitization of endometrial cancer cells to apoptosis was reported when GRP78 expression was downregulated in these cells by the use of specific siRNAs [36]. Another major finding of our study is that metformin could inhibit the Wnt/β-catenin pathway. Metformin inhibited, indeed, β-catenin expression at both the mRNA and protein level (Figure 5A–C and Supplementary Materials Figure S2). As a consequence, β-catenin transcriptional activity was also reduced, as confirmed by the reduction in activity of a TCF/LEF promoter (Figure 5D). Again, these effects appeared to be AMPK-independent, since CC was unable to prevent the inhibition of β-catenin protein expression induced by metformin (Figure 5B and Supplementary Materials Figure S2). Inhibition of the Wnt/β-catenin pathway by metformin has been described in different cancer cell types and particularly in colon carcinoma cells [37,38,39]. Park et al. [39] reported that β-catenin inhibition appeared to be dependent from AMPK activation. The discrepancy of our findings might be explained by the different cellular context regulating endometrial cancer cells. Furthermore, we found that metformin might affect also β-catenin protein stability, since we observed a reduction in GSK3-β phosphorylation in the Ser 9 residue (Figure 6). The accumulation of β-catenin inside the cells is, indeed, largely dependent from GSK3-β phosphorylation status and Ser 9 phosphorylation, inactivating GSK3-β, favors β-catenin stability and accumulation [48]. The decline in the inhibitory serine-phosphorylation of GSK3-β following metformin treatment might be also a consequence of the reduction in Akt phosphorylation, since GSK3 is a well-known substrate of Akt. It has been reported, indeed, that AMPK activating agents, such as phenformin and AICAR, cause dephosphorylation of Akt and glycogen synthase kinase-3 in neuroblastoma cells [49]. However, CC was not able to prevent Akt and GSK3-β dephosphorylation. Thus, metformin might promote a hyperactive state of GSK3-β in endometrial cancer cells, resulting in a further decrease in β-catenin protein and a drop in the transcription of TCF/LEF target genes, including the cell-cycle regulatory genes cyclin D1 and c-myc. In summary, we describe new molecular mechanisms not dependent on the activation of AMPK, potentially involved in metformin anti-tumor activity in endometrial cancer cells (Figure 7). Further investigations ongoing in our laboratories will clarify whether the UPR and the Wnt/β-catenin pathways might be causally connected. This might inform new therapeutic approaches for the treatment of endometrial cancer.

## Data Availability

All data are available upon request from the corresponding author.

## Supplementary Materials

The following are available online at https://www.mdpi.com/article/10.3390/cells10051067/s1, Figure S1: CC does not affect AKT phosphorylation and GRP78 expression in Ishikawa and AN3CA cells; Figure S2: Metformin decreases β-catenin expression in both cytosolic and nuclear extracts of Ishikawa and AN3CA cells.
