# Supplementary material for: Metformin Dysregulates the Unfolded Protein Response and the WNT/β-Catenin Pathway in Endometrial Cancer Cells through an AMPK-Independent Mechanism
Source: Cells. 2021 Apr 30;10(5):1067. doi: 10.3390/cells10051067 (PMC8147131; doi:10.3390/cells10051067)
Supplement: Supplementary file 1 [file cells-10-01067-s001.zip › cells-1143851-supplementary.pdf]

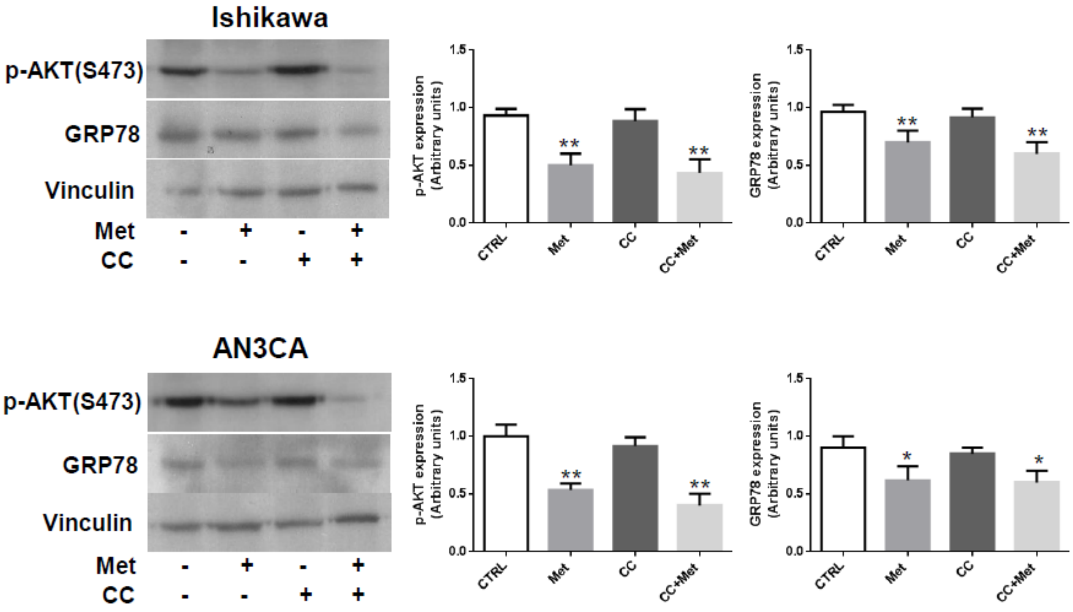

Figure S1. CC does not affect AKT phosphorylation and GRP78 expression in Ishikawa and AN3CA cells. Ishikawa and AN3CA cells were treated or not for 24 hours with 5 mM metformin or 10 µM CC or pretreated for 1 hour with 10 µM CC followed by treatment with 5 mM metformin. Total cellular proteins were extracted and Western Blot experiments were performed, as described in the "Materials and Methods" section, using antibodies against p-AKT (S473) or GRP78. Data represent the mean ± SD of three independent experiments. * $p<0.1$; ** $p<0.05$.

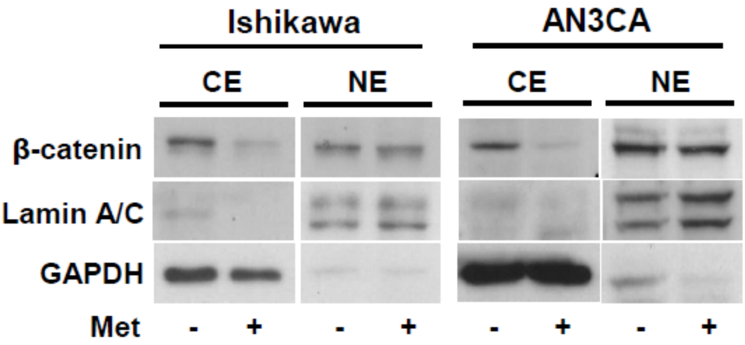

Figure S2. Metformin decreases β-catenin expression in both cytosolic and nuclear extracts of Ishikawa and AN3CA cells. Ishikawa and AN3CA cells were treated for 24 hours with 5 mM metformin. Following cell fractionation, as described in the "Materials and Methods" section, equal amounts of cytoplasmic (CE) and nuclear (NE) extracts were subjected to immunoblotting using anti-β-catenin, anti-lamin A/C, and anti-GAPDH antibodies.

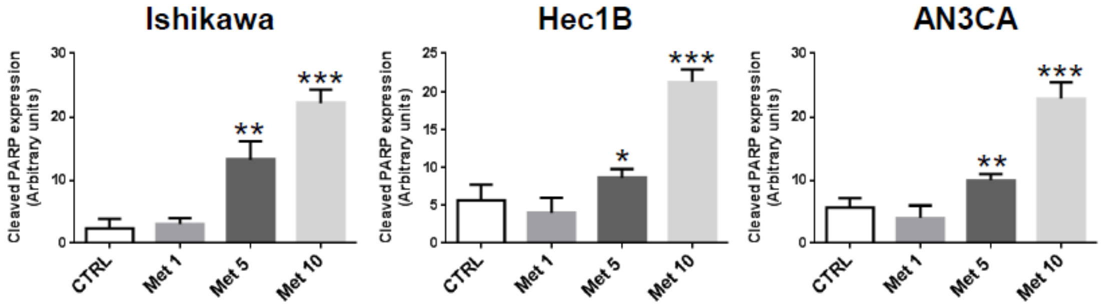

Figure S3. Graphs show densitometric analysis of cleaved PARP (Figure 1D). * indicates a *p* value<0.1; ** indicates a *p* value<0.01; *** indicates a *p* value<0.001
